# Temperament, personality and decision-making accuracy in handball referees

**DOI:** 10.1371/journal.pone.0353879

**Published:** 2026-07-16

**Authors:** Jacek Świdwa, Magdalena Król-Zielińska, Adam Kantanista

**Affiliations:** Department of Physical Education and Lifelong Sports, Poznan University of Physical Education, Poznań, Poland; Universidade Europeia, Lisboa, PORTUGAL

## Abstract

Accurate decision-making is essential for effective handball officiating, yet little is known about how stable psychological traits relate to referees’ decision-making accuracy. This study examined whether temperament and personality characteristics are associated with decision-making accuracy in handball referees. Forty-one officials from three competition levels (Second League, First League, Superleague) completed the FCB-TI and NEO-FFI inventories. Decision-making accuracy was assessed using a video-based test of game situations scored against expert judgments. Sensory Sensitivity showed a positive bivariate correlation with decision-making accuracy (*r* = 0.32, *p* < 0.05). Multiple regression analysis revealed that competitive level accounted for 24% of the variance in decision-making accuracy (*R^2^* = 0.24, *p* = 0.001). Adding Sensory Sensitivity increased the explained variance to 30%, although its unique contribution did not reach statistical significance. Activity, as a temperament trait, showed different associations with decision-making accuracy across competition levels. No significant relationships were observed between Big Five personality traits and decision-making accuracy. These findings suggest that, among the variables examined, competitive level was the main factor associated with decision-making accuracy, while selected temperament traits, particularly Sensory Sensitivity and, to a lesser extent, Activity, may also be relevant to this outcome.

## Introduction

Officiating in team sports such as handball requires rapid processing of dynamic visual information and accurate decision-making under time pressure [1–4]. Referees also operate under evaluation from players, coaches, and spectators, and decision errors in critical situations may influence match outcomes [5–7]. Decision-making accuracy is commonly regarded as one of the main indicators of refereeing expertise [3,8]. Effective officiating requires physical preparation and knowledge of the rules, as well as attentional control, emotional regulation, and the ability to function under pressure [9–11].

Referees must combine current visual information with knowledge stored in long-term memory and expectations about how a situation may develop [12–14]. Previous research has therefore focused mainly on perceptual–cognitive expertise, including visual search, cue recognition, and the selection of relevant information [15–20]. These skills are important because decision-making accuracy depends on identifying relevant cues and selecting an appropriate response [21,22]. Plessner et al. [23] proposed that refereeing decisions can be understood through a multiple-cue learning approach. According to this approach, referees learn which situational cues are relevant and how much weight should be assigned to them. Decision-making accuracy therefore depends not only on observing the available information, but also on how the referee interprets and combines multiple cues. This approach may help explain why referees can reach different decisions when viewing the same situation. However, perceptual–cognitive expertise alone may not fully explain differences in decision-making accuracy among referees operating at similar competitive levels.

Refereeing decisions are also affected by the competitive environment. Nevill et al. [24] found that crowd noise influenced football referees’ evaluations of recorded match incidents and reduced the number of decisions made against the home team. Dohmen et al. [25] using data from professional football matches, also reported home-team advantages in decisions concerning stoppage time and selected goal- and penalty-related situations. Studies conducted during matches played without spectators similarly suggest that the absence of crowd pressure may reduce some forms of home-team bias [24,26]. At the same time, the absence of spectators may alter referees’ stress, motivation, emotional arousal, and professional engagement [27–30]. These studies show that refereeing decisions are sensitive to social and situational conditions. However, they do not explain why referees may respond differently to similar environmental demands. This suggests that individual psychological characteristics should also be considered.

Research on the psychological functioning of referees has examined self-efficacy, coping with external stressors, psychological preparation, stress tolerance, and mental skills [31–38]. Recent studies have also shown that psychological and physical load, including mental fatigue, may influence referees’ decision-making performance [39,40]. Referee self-efficacy refers to confidence in one’s ability to perform officiating duties effectively and has been associated with coping, emotional control, and professional functioning among sports officials [32,33,37]. Although self-efficacy may partly reflect how referees evaluate their own ability to meet task demands, it is conceptually different from relatively stable personality and temperament traits. Moreover, evidence regarding the relationship between such psychological characteristics and objectively measured decision-making accuracy remains limited.

Examining the role of individual psychological characteristics in referees’ decision-making requires a performance-based measure of decision-making accuracy. Video-based tests allow referees to make decisions about the same match situations under standardized presentation and response conditions and have been shown to differentiate between officials of different performance levels [41].

Personality is one possible source of relatively stable differences between referees. Within the Five-Factor Model, personality is described in terms of Neuroticism, Extraversion, Openness to Experience, Agreeableness, and Conscientiousness [42,43]. Some personality traits may also be relevant to refereeing. Conscientiousness may support preparation, responsibility, and adherence to professional standards. Emotional stability may help referees cope with criticism and pressure, whereas extraversion may support communication and game management. Dodt et al. [44] found that expert handball referees reported higher extraversion, agreeableness, and conscientiousness and lower negative emotionality than the general population. Similar patterns were also observed among amateur handball referees, although only limited differences were found between amateur and expert officials. The perceived importance of personality characteristics has also been examined in the sport environment but from the perspective of handball players and coaches [45]. This research indicates that confidence, calmness, communication, leadership, reliability, and conscientiousness-related characteristics are viewed as important for effective refereeing. These characteristics appear particularly relevant to game management, interaction with players and coaches, and maintaining authority during competition.

Broader sport research provides only indirect evidence, because associations between personality and athletic performance do not necessarily translate into referees’ decision-making accuracy. Personality may influence broader aspects of officiating, such as emotional control, communication, preparation, or professional behavior, without necessarily determining the correctness of decisions made within a few seconds of observing a game situation. Direct evidence linking Big Five traits with objectively assessed refereeing decision-making accuracy remains limited [46–48].

From a psychological perspective temperament may be particularly relevant to decision-making. According to the Regulative Theory of Temperament, temperament refers to biologically based characteristics of behavior that regulate responses to stimulation [49,50]. The theory distinguishes six traits: Sensory Sensitivity, Emotional Reactivity, Briskness, Perseveration, Endurance, and Activity. These traits may be relevant to refereeing in different ways. Sensory Sensitivity refers to the ability to respond to low-intensity stimuli and may be important for detecting subtle situational cues. Briskness describes the tendency to respond quickly and adjust behavior to changing conditions. Endurance concerns the ability to function under intensive or prolonged stimulation. Emotional Reactivity reflects the intensity of emotional responses and may be relevant when referees are exposed to criticism and social pressure. Activity refers to the tendency to seek highly stimulating situations, whereas perseveration describes the continuation of a response after the stimulus has ceased [49,50].

The potential role of temperament is consistent with the demands of refereeing. Referees must detect relevant cues, shift attention, regulate arousal, and respond within a limited time. Temperament concerns formal characteristics of behavior and the regulation of stimulation, whereas personality refers to broader patterns of motivation, interpersonal behavior, and self-regulation. For this reason, temperament may be more closely related to performance under time-constrained decision-making conditions, whereas personality may be more relevant to broader aspects of professional functioning, preparation, and game management. However, this distinction has not been sufficiently examined among sports officials.

Direct evidence on temperament in referees remains limited. Previous studies in basketball and football referees have suggested that psychological preparedness, stress resistance, coping strategies, and emotional functioning may be relevant to effective officiating and refereeing styles [51,52]. However, these studies used traditional temperament typologies or broader psychological indicators rather than the Regulative Theory of Temperament, and they did not assess objectively measured decision-making accuracy. Findings from athlete populations provide only indirect support, suggesting that temperamental characteristics may be related to functioning under different forms and levels of stimulation, including pain sensitivity in combat sport athletes, participation in extreme and high-risk sports, differences between track-and-field specializations, and performance in simulated football game situations [53–56].

Therefore, it remains unclear whether temperament traits defined within the Regulative Theory of Temperament are associated with the decision-making accuracy of referees.

Taken together, previous studies indicate that refereeing decisions are related to perceptual–cognitive expertise, competitive experience, crowd pressure, self-efficacy, coping, mental skills, and temporary physical and psychological load. However, the role of relatively stable psychological characteristics in objectively assessed decision-making accuracy remains less clear.

The central research question was whether temperament and personality traits were associated with decision-making accuracy after competitive level was considered. Therefore, the present study examined the relationships between competitive level, temperament, personality, and decision-making accuracy among handball referees. We expected that decision-making accuracy would be positively associated with temperament traits and that temperament would account for a greater proportion of variance in decision-making accuracy than personality traits. In addition, it was hypothesized that referees officiating at higher competitive levels would demonstrate greater decision-making accuracy.

## Methods

### Participants

The study included 41 active handball referees (30 men and 11 women) registered with the Polish Handball Federation, representing approximately 20% of the national referee population. The participants officiated at three competitive levels: Superleague (elite level), First League, and Second League in Poland. The referees had an average age of 29.71 ± 7.16 years (men: 30.33 ± 7.67; women: 28.00 ± 5.49, *p* = 0.362).

All participants were recruited from August 1, 2021, to March 31, 2022. Written informed consent was obtained from all individuals prior to data collection. The study received approval from the Ethics Committee of the Poznan University of Medical Sciences (Approval number: KB/357/21) and adhered to the ethical standards of the Declaration of Helsinki.

### Procedure and test protocol

Prior to the decision-making accuracy task, all participants completed a brief survey assessing demographic information, refereeing experience, and handball-related background. Testing was conducted individually in a controlled laboratory setting, with only the participant and a trained evaluator present.

Participants were tested individually,positioned in front of a projection screen and asked to evaluate 30 video-based handball match situations, each depicting a rule-relevant event (e.g., foul, contact violation, illegal action). Each video clip lasted 5 seconds and was presented only once, with no possibility of replay. After each clip, participants had 5 seconds to select the decision they considered correct. Participants were instructed to respond as they would during an actual match, balancing decision-making accuracy with realistic time constraints. All responses were recorded and later compared with an expert reference key (developed by one technical delegate and two international referees) which aligned with the official rules of handball, to determine individual decision-making accuracy.

Prior to data collection, each of the 30 match situations was independently analyzed and scored by the panel, and all decisions were unanimous, providing a clear and objective benchmark for evaluating participants’ responses.

In addition to the laboratory task, all referees completed an online temperament and personality questionnaire distributed via the internal mailing system of the Polish Handball Federation. The survey included an electronic informed consent form and was fully completed by all participants.

### Measures

The NEO Five-Factor Inventory (NEO-FFI) was used to evaluate the personality of referees. It is a standardized instrument designed to assess the five core dimensions of personality defined within the Five-Factor Model (FFM): Neuroticism, Extraversion, Openness to Experience, Agreeableness, and Conscientiousness. This shortened version of the NEO Personality Inventory-Revised (NEO-PI-R) consists of 60 self-report items and requires approximately 10–15 minutes to complete [42,43]. Participants respond on a five-point Likert scale ranging from 1 (“strongly disagree”) to 5 (“strongly agree”).

The NEO-FFI demonstrates strong psychometric properties, including high internal consistency and solid construct validity, supported by correlations with observer ratings, behavioral measures, genetic research on trait heritability, and convergence with other established personality assessments [42,43]. Cross-cultural applications of the scale further confirm the structural stability of the FFM and the utility of the NEO-FFI as an efficient and reliable tool for personality assessment. In the present sample, all NEO-FFI scales demonstrated satisfactory to high internal consistency. Cronbach’s alpha coefficients were 0.85 for Neuroticism, 0.78 for Extraversion, 0.74 for Openness to Experience, 0.76 for Agreeableness, and 0.77 for Conscientiousness. These values were consistent with the reliability coefficients reported in Polish normative and adaptation studies [43].

Temperamental traits were assessed using the Formal Characteristics of Behavior–Temperament Inventory (FCB-TI) [49], developed in accordance with principles for constructing rating scales [50] and grounded in the Regulative Theory of Temperament (RTT). The questionnaire comprises 120 items measuring six biologically based traits that characterize individual styles of reacting and functioning: Emotional Reactivity, Sensory Sensitivity, Perseveration, Endurance, Briskness, and Activity.

Psychometric analyses indicated that the FCB-TI demonstrates satisfactory theoretical validity and measurement quality. In the present sample, all FCB-TI scales demonstrated satisfactory to high internal consistency. Cronbach’s alpha coefficients were 0.85 for Emotional Reactivity, 0.77 for Sensory Sensitivity, 0.77 for Perseveration, 0.88 for Endurance, 0.71 for Briskness, and 0.87 for Activity. These values were consistent with the reliability coefficients reported in Polish normative and adaptation studies [49].

To provide an additional norm-referenced descriptive interpretation of the psychological test results, raw scores from the NEO-FFI and FCB-TI were also transformed into standardized categories according to the Polish normative procedures. Sten and stanine scores were classified into low, average, and high categories. These categorical scores were used only to describe the psychological profile of referees with lower and higher decision-making accuracy. For this purpose, participants were divided according to the third quartile of decision-making accuracy, with referees scoring at or above Q3 (65% correct responses) classified as the higher-decision-making accuracy group. This analysis was descriptive in nature and was not treated as the main inferential approach.

### Statistical analysis

Statistical analyses were performed using Statistica 13.3 (TIBCO Software Inc.). Descriptive statistics, including means and standard deviations, were calculated for all study variables. Prior to the main analyses, assumptions of normality, homogeneity of variance, and absence of extreme outliers were evaluated using the Shapiro–Wilk test, Levene’s test, and inspection of the data distribution.

An a priori power analysis was conducted using G*Power 3.1 to estimate the minimum sample size required for the planned regression analysis. The analysis was specified for multiple regression with an alpha level of 0.05, statistical power of 0.95, and an expected effect size of f² = 0.43, corresponding to *R*² = 0.30. The required minimum sample size was 28 participants. The final sample size of 41 referees therefore exceeded the minimum required for the main regression model.

To assess gender differences in decision-making accuracy, independent-samples t-tests were used, whereas differences between competitive levels were examined using one-way ANOVA. Tukey’s post hoc test was used for pairwise comparisons when the ANOVA result was significant. Relationships between temperament traits, personality traits, and decision-making accuracy were assessed using Pearson correlation coefficients or, when appropriate, Spearman correlation coefficients.

Multiple regression was used to determine the extent to which temperament, personality traits, and competition level explained variance in decision-making accuracy. Statistical significance was set at *p* < 0.05.

Standardized norm-referenced scores were used to provide a descriptive interpretation of the distribution of temperament and personality traits.

## Results

The overall mean decision-making accuracy score was 17.51 ± 3.17 (men: 17.80 ± 3.40; women: 16.73 ± 2.37; *p* = 0.343). No significant gender difference was found in decision-making accuracy. Referees differed significantly in decision-making accuracy across competitive levels (*F* = 6.62, *p* < 0.05). Post hoc analysis revealed that referees from the Superleague achieved significantly higher decision-making accuracy scores (19.77 ± 2.77) than referees from the First League (17.00 ± 3.08; *p* = 0.042) and the Second League (16.00 ± 2.56; *p* = 0.003). The difference between First League and Second League referees was not significant (*p* = 0.618). These findings indicate that the highest-level referees demonstrated greater decision-making accuracy than referees from lower competitive levels. Descriptive statistics of temperament and personality traits in referees from different leagues are presented in Table 1.

**Table 1:** Descriptive statistics of temperament and personality traits in referees from different leagues.

Variable	Level of league			
	Total	Second	First	Super
	*M* ± *SD* *n* = 41	*M* ± *SD* *n = 15*	*M* ± *SD* *n = 13*	*M* ± *SD* *n = 13*
**Decision-making accuracy**	17.51 ± 3.17	16.00 ± 2.56	17.00 ± 3.08	19.77 ± 2.77
**Temperament**				
Briskness	17.17 ± 2.73	15.93 ± 2.94	18.08 ± 2.10	17.69 ± 2.72
Perseveration	11.26 ± 4.12	11.33 ± 4.67	10.46 ± 4.25	12.00 ± 3.46
Sensory Sensitivity	14.19 ± 3.74	13.67 ± 3.98	13.69 ± 4.59	15.31 ± 2.29
Emotional reactivity	5.41 ± 4.40	6.07 ± 5.30	3.62 ± 2.10	6.46 ± 4.72
Endurance	13.58 ± 4.96	12.73 ± 5.76	15.38 ± 3.38	12.77 ± 5.18
Activity	12.58 ± 4.85	10.60 ± 4.61	13.92 ± 4.59	13.54 ± 4.98
**Personality**				
Neuroticism	14.60 ± 8.15	16.20 ± 8.66	12.00 ± 7.42	15.38 ± 8.24
Extraversion	32.21 ± 6.56	29.73 ± 6.34	34.62 ± 5.80	32.69 ± 7.00
Openness to Experience	23.85 ± 6.69	22.73 ± 6.22	23.08 ± 5.16	25.92 ± 8.42
Agreeableness	29.31 ± 6.34	28.13 ± 5.97	29.62 ± 6.20	30.38 ± 7.15
Conscientiousness	37.70 ± 5.50	36.27 ± 6.34	38.69 ± 5.39	38.38 ± 4.56

Note. *M* = mean; *SD* = standard deviation; *n* = number of participants.

Correlation coefficients between decision-making accuracy and temperament and personality traits in handball referees are presented in Table 2. In the total sample, among temperament traits, Sensory Sensitivity was the only variable significantly associated with decision-making accuracy (r = 0.32, *p* = 0.043). No significant associations were found for Briskness, Perseveration, Emotional Reactivity, Endurance, or Activity in the total sample (*p* > 0.05). In the analyses conducted separately within competitive levels, Activity, as a temperament trait, showed different associations with decision-making accuracy across league groups. Among Second League referees, Activity was negatively associated with decision-making accuracy (*r* = −0.59, *p* < 0.05), whereas among First League referees, Activity was positively associated with decision-making accuracy (*r* = 0.59, *p* < 0.05). In the Superleague group, the association was negative but did not reach statistical significance (*r* = −0.47, *p* > 0.05). Other temperament traits did not show significant within-league associations with decision-making accuracy.

**Table 2:** Correlation coefficients between decision-making accuracy and temperament and personality traits in handball referees.

Variable	Decision-making accuracy			
	Level of league			
	Total	Second	First	Super
**Temperament**				
Briskness	*r*_*S*_ = 0.22	*r*_*P*_ = −0.23	*r*_*S*_ = 0.25	*r*_*S*_ = 0.37
Perseveration	*r*_*P*_ = 0.15	*r*_*P*_ = 0.20	*r*_*P*_ = 0.25	*r*_*P*_ = −0.17
Sensory Sensitivity	***r***_***P***_ = 0.32*	*r*_*P*_ = 0.01	*r*_*P*_ = 0.48	*r*_*P*_ = 0.25
Emotional Reactivity	*r*_*S*_ = 0.13	*r*_*P*_ = 0.28	*r*_*P*_ = 0.06	*r*_*P*_ = −0.10
Endurance	*r*_*S*_ = −0.19	*r*_*P*_ = −0.33	*r*_*P*_ = −0.14	*r*_*P*_ = 0.12
Activity	*r*_*P*_ = −0.02	***r***_***P***_ = -0.59*	***r***_***P***_ = 0.59*	*r*_*P*_ = −0.47
**Personality**				
Neuroticism	*r*_*P*_ = 0.12	*r*_*P*_ = 0.19	*r*_*P*_ = 0.42	*r*_*P*_ = −0.18
Extraversion	*r*_*P*_ = −0.05	*r*_*P*_ = −0.28	*r*_*P*_ = 0.14	*r*_*P*_ = −0.27
Openness to Experience	*r*_*P*_ = 0.03	*r*_*P*_ = −0.35	*r*_*P*_ = 0.26	*r*_*P*_ = −0.14
Agreeableness	*r*_*P*_ = −0.10	*r*_*P*_ = −0.14	*r*_*P*_ = −0.21	*r*_*P*_ = −0.23
Conscientiousness	*r*_*P*_ = −0.23	*r*_*P*_ = −0.28	*r*_*P*_ = −0.33	*r*_*P*_ = −0.50

Note. *M* = mean; *SD* = standard deviation; *r*_*P*_ = Pearson correlation coefficient; *r*_*S*_ = Spearman correlation coefficient; * *p* < 0.05.

Regarding the Big Five personality traits, no significant associations with decision-making accuracy were found either in the total sample or within any competitive level subgroup (*p* > 0.05).

Multiple regression analysis was conducted to examine the contribution of competitive level and Sensory Sensitivity to decision-making accuracy. In Step 1, competitive level accounted for 24% of the variance in decision-making accuracy (*R*^2^ = 0.24, *F*(1, 39) = 12.39, *p* = 0.001), with a significant standardized effect (β = 0.49, *p* = 0.001). Adding Sensory Sensitivity in Step 2 increased the explained variance to 30% (*R^2^* = 0.30, *F*(2, 38) = 7.98, *p* = 0.001), with a change in explained variance of Δ*R^2^* = 0.06. Although the effect of Sensory Sensitivity did not reach conventional statistical significance (β = 0.24, *p* = 0.094), the result may indicate a possible contribution of this trait to decision-making accuracy beyond competitive level.

Exploratory league-specific regression models were also conducted for Activity, based on the significant within-league correlations observed in the previous analysis. In the Second League group, Activity negatively predicted decision-making accuracy (*R*² = 0.34, β = −0.59, *F* = 6.81, *p* = 0.022). In the First League group, Activity positively predicted decision-making accuracy (*R*² = 0.35, β = 0.59, *F* = 5.84, *p* = 0.034). The results are presented in Table 3.

**Table 3:** Regression models predicting decision-making accuracy in handball referees.

Variable	*R* ^*2*^	β	*F*	*p-*value
Model 1: Total *n* = 41				
Step 1	0.24		12.39	0.001
Level of league		0.49		0.001
Step 2	0.30		7.98	0.001
Level of league		0.45		0.003
Sensory Sensitivity		0.24		0.094
Model 2: Second league *n* = 15	0.34		6.81	0.022
Activity		−0.59		0.022
Model 3: First league *n* = 13	0.35		5.84	0.034
Activity		0.59		0.034

Note. *n* = number of participants.

A supplementary descriptive analysis based on Polish normative score categories was conducted to characterize the distribution of personality and temperament profiles according to decision-making accuracy group. As shown in Table 4, the distribution of low, average, and high standardized scores was generally similar between the lower- and higher-accuracy groups. The most visible descriptive difference concerned Sensory Sensitivity: referees in the higher decision-making accuracy group were less frequently classified as low and more frequently classified as average in this trait. A higher proportion of high scores in Briskness and Activity was also observed in the higher decision-making accuracy group.

**Table 4:** Distribution of standardized personality and temperament traits according to decision-making accuracy group.

Trait	Category	Lower-accuracy group (%)*n* = 29	Higher-accuracy group (%)*n* = 12	Total sample (%)*n* = 41
Neuroticism	Low	48.3	41.7	46.3
	Average	44.8	58.3	48.8
	High	6.9	0.0	4.9
Extraversion	Low	3.4	0.0	2.4
	Average	55.2	50.0	53.7
	High	41.4	50.0	43.9
Openness to Experience	Low	41.4	33.3	39.0
	Average	51.7	50.0	51.2
	High	6.9	16.7	9.8
Agreeableness	Low	17.2	33.3	22.0
	Average	58.6	50.0	56.1
	High	24.1	16.7	22.0
Conscientiousness	Low	3.4	0.0	2.4
	Average	51.7	33.3	46.3
	High	44.8	66.7	51.2
Emotional Reactivity	Low	55.2	66.7	58.5
	Average	37.9	33.3	36.6
	High	6.9	0.0	4.9
Sensory Sensitivity	Low	41.4	8.3	31.7
	Average	41.4	75.0	51.2
	High	17.2	16.7	17.1
Perseveration	Low	27.6	16.7	24.4
	Average	55.2	75.0	61.0
	High	17.2	8.3	14.6
Briskness	Low	6.9	8.3	7.3
	Average	41.4	16.7	34.1
	High	51.7	75.0	58.5
Activity	Low	13.8	8.3	12.2
	Average	44.8	33.3	41.5
	High	41.4	58.3	46.3
Endurance	Low	6.9	8.3	7.3
	Average	37.9	41.7	39.0
	High	55.2	50.0	53.7

*Note. n* = number of participants; NEO-FFI traits were classified using sten norms and FCB-TI traits using stanine norms; Categories were based on Polish test norms.

## Discussion

The present study examined whether temperament and personality traits were associated with decision-making accuracy in handball referees after competitive level was considered. To our knowledge, this is the first study to integrate measures of temperament and personality with objectively assessed decision-making accuracy in the context of sports officiating. The results provide several important insights, extending current theoretical understanding in this still-developing area of sport psychology while also offering practical implications for referee training programs.

Among the temperament traits, Sensory Sensitivity showed the strongest bivariate association with decision-making accuracy. However, its unique contribution did not reach statistical significance after competitive level was included in the regression model. Referees who were more sensitive to subtle sensory cues achieved higher levels of decision-making accuracy, a pattern that was particularly evident at the intermediate level of competition. This finding is consistent with the assumptions of the Regulative Theory of Temperament [50], which proposes that heightened sensitivity to low-intensity stimuli may facilitate more efficient intake of information in dynamic environments. Sensory Sensitivity may be relevant to the detection of subtle situational cues, although its independent contribution requires confirmation in larger samples [3]. Perceptually well-attuned officials may also experience greater confidence in their judgments, which may further support decision-making accuracy through increased refereeing self-efficacy [32,35]. As previous research has shown, effective refereeing depends on a combination of game knowledge, decision-making skills, psychological competencies, strategic abilities, communication and match-control skills, and physical fitness [34,36]. In the present study, the strongest association between Sensory Sensitivity and decision-making accuracy was observed among First League referees. This finding suggests that this stage of professional development may be particularly important, as biological predispositions appear to support the refinement of key competencies before decision-making processes become more automated.

With respect to Activity, different associations with decision-making accuracy were observed across competitive levels, a pattern that may be interpreted considering the goodness-of-fit framework [57]. Among Second League referees, higher Activity levels were associated with lower decision-making accuracy, suggesting that elevated energetic arousal may promote impulsive or premature judgments during the early stages of refereeing development. In contrast, within the First League group, Activity positively predicted decision-making accuracy. This finding indicates that once basic refereeing skills become consolidated, higher arousal may support attentional mobilization and cognitive readiness. At the elite level (Superleague), this relationship did not reach statistical significance. One possible interpretation is that the adaptive value of heightened Activity may change when perceptual–cognitive demands are already very high and decision processes are further automated. Overall, the functional role of Activity appears to depend on competitive context and to change across successive stages of referees’ career development, consistent with our assumptions and earlier research on decision-making among sports officials [3,11,58]. However, these subgroup findings should be interpreted with caution because of the small number of referees in each competitive-level group.

Competitive level emerged as the main factor associated with decision-making accuracy among the variables examined. Referees officiating at higher competitive levels achieved significantly better results than those operating at lower levels. This difference may reflect repeated exposure to increasingly demanding match situations, more effective use of relevant perceptual cues, and more automated decision-making processes [3,8,23]. However, these mechanisms were not directly assessed in the present study. Competitive level explained approximately one quarter of the variance in decision-making accuracy, indicating that substantial individual differences remained unexplained. In real competitive settings, the expression of temperamental predispositions may also be shaped by relational and contextual factors. During actual handball matches, cooperation between the two referees plays a crucial role in the decision-making process. Previous research suggests that the length of shared officiating experience, gradual improvement in interpersonal relationships, and mutual agreement between referees serve as additional important predictors of officiating pair effectiveness [32]. The literature consistently emphasizes that cooperation, open communication, mutual support, trust, and respect form the foundation of effective teamwork among officials. By contrast, strained or conflictual relationships between co-referees are frequently identified as major sources of occupational stress and may contribute to the development of burnout [32,58,59]. At the elite level of competition, officiating pairs are often composed of referees who, despite extensive individual experience, have also worked together with the same partner across multiple seasons. Such continuity may enhance communication quality, strengthen mutual trust, and facilitate the progressive automation of joint assessments of on-court incidents. These factors were not measured in the present study and should be considered in future research. In contrast to findings typically reported in athlete samples, where Conscientiousness is often identified as a positive predictor of sport performance [46,48], in our study none of the Big Five traits was significantly associated with decision-making accuracy. Previous studies suggest that personality may be more relevant to communication, game management, responsibility, and adaptation to the refereeing role than to the correctness of individual decisions [60]. The findings are consistent with the view that competitive level provides the main context for decision performance, while temperament may show more specific and level-dependent associations. Broad personality traits were not directly associated with decision-making accuracy in the present sample. From this perspective, refereeing emerges as a unique domain in which perceptual, emotional, and dispositional processes are regulated simultaneously under strong time constraints.

The findings do not provide sufficient support for using all temperament traits as independent selection criteria. However, if replicated, information about individual differences in stimulation processing could contribute to individualized training, for example by varying time pressure, perceptual complexity, distraction, and emotional load [11,22,54,57].

### Theoretical and practical implications

Our results suggest that Sensory Sensitivity and Activity, as temperament traits, may contribute to referees’ decision-making accuracy, particularly in relation to how referees detect situational cues, tolerate stimulation, and regulate their responses under demanding match conditions. The subgroup results for Activity may further indicate that the role of temperament differs depending on the stage of refereeing expertise. At different competitive levels, the tendency to seek or tolerate high levels of stimulation may have different functional meanings: it may support adaptive engagement and readiness in some referees but may also be associated with excessive stimulation or less efficient regulation in others. From a theoretical perspective, these findings support the view that officiating decisions may be shaped by the interaction between perceptual–cognitive expertise, situational demands, and some individual psychological characteristics. From a practical perspective, the results highlight the potential value of considering individual differences in referee education and psychological preparation, particularly in relation to cue perception, arousal regulation, and coping with match-related pressure. However, these findings should not be interpreted as only supporting temperament-based referee selection. They suggest directions for future research on individualized development programs for referees.

### Limitations and future research directions

Several limitations should be noted. Although the sample represented approximately 20% of central-level referees registered with the Polish Handball Federation, the number of participants within each competitive-level subgroup was limited. Therefore, subgroup findings, particularly those concerning Activity, should be treated as exploratory. In addition, the cross-sectional design does not allow conclusions about causality or changes in decision-making accuracy over time. Decision-making accuracy was assessed under standardized laboratory conditions, which ensured comparability across participants but did not fully reproduce the physical, social, and emotional demands of actual competition. Future studies should replicate these findings in larger and more diverse samples and use more naturalistic or hybrid designs, including virtual-reality tasks, eye-tracking, match-tracking technologies, and psychophysiological indicators such as heart-rate variability. Such approaches may help clarify how temperament, perceptual–cognitive expertise, fatigue, crowd pressure, match importance, and cooperation between referee partners jointly shape decision-making accuracy.

## Conclusions

Decision-making accuracy was primarily associated with competitive level among the variables included in this study. Sensory Sensitivity was positively related to decision-making accuracy in the bivariate analysis. This association was not statistically significant after competitive level was included in the model. No significant associations were found between Big Five personality traits and decision-making accuracy. Activity showed different associations with decision-making accuracy across competitive levels, suggesting that the functional role of temperament may vary depending on the stage of refereeing expertise, although these level-specific findings require replication. Taken together, the results indicate that competitive level remains the strongest factor associated with decision-making accuracy, while selected temperament traits may help explain individual differences in how referees process situational cues and regulate stimulation under decision-making demands. This study extends research on sports officiating by integrating temperament, personality, and objectively measured decision-making accuracy within the same group of handball referees.

## References

[1] Cuskelly G, Smith C, Rynne J. Predicting the retention of sports officials: the influence of stress, commitment and perceived organisational support. In: European Association for Sport Management. 2008.

[2] Gorostiaga EM, Granados C, Ibañez J, González-Badillo JJ, Izquierdo M. Effects of an entire season on physical fitness changes in elite male handball players. Med Sci Sports Exerc. 2006;38(2):357–66. doi: 10.1249/01.mss.0000184586.74398.03 16531907

[3] Helsen WF, MacMahon C, Spitz J. Decision making in match officials and judges. In: Williams AM, Jackson RC, editors. Anticipation and decision making in sport. London: Routledge. 2019. p. 250–66.

[4] Schrödter R, Schwarting A, Fasold F, Schul K, Klatt S. The Relevance of General Spatial Anticipation Skills for Basketball Referees. Applied Sciences. 2023;13(5):2991. doi: 10.3390/app13052991

[5] Anderson KJ, Pierce DA. Officiating bias: the effect of foul differential on foul calls in NCAA basketball. J Sports Sci. 2009;27(7):687–94. doi: 10.1080/02640410902729733 19424898

[6] Mascarenhas DRD, Smith NC. Developing the performance brain: decision making under pressure. In: Collins D, Button A, Richards H, editors. Performance psychology: a practitioner’s guide. Edinburgh: Churchill Livingstone Elsevier; 2011. p. 245–67.

[7] Guillén F, Feltz DL. A conceptual model of referee efficacy. Front Psychol. 2011;2:25. doi: 10.3389/fpsyg.2011.00025 21713174 PMC3111226

[8] Weinberg RS, Richardson PA. Psychology of officiating. Champaign, IL: Leisure Press; 1990.

[9] MacMahon C, Helsen WF, Starkes JL, Weston M. Decision-making skills and deliberate practice in elite association football referees. J Sports Sci. 2007;25(1):65–78. doi: 10.1080/02640410600718640 17127582

[10] Spitz J, Put K, Wagemans J, Williams AM, Helsen WF. Visual search behaviors of association football referees during assessment of foul play situations. Cogn Res Princ Implic. 2016;1(1):12. doi: 10.1186/s41235-016-0013-8 28180163 PMC5256438

[11] Raab M, Avugos S, Bar-Eli M, MacMahon C. The referee’s challenge: a threshold process model for decision making in sport games. Int Rev Sport Exerc Psychol. 2020;14(1):208–28. doi: 10.1080/1750984x.2020.1783696

[12] Mann DTY, Williams AM, Ward P, Janelle CM. Perceptual-cognitive expertise in sport: a meta-analysis. J Sport Exerc Psychol. 2007;29(4):457–78. doi: 10.1123/jsep.29.4.457 17968048

[13] Ericsson KA, Kintsch W. Long-term working memory. Psychol Rev. 1995;102(2):211–45. doi: 10.1037/0033-295x.102.2.211 7740089

[14] van Biemen T, van Zanten TF, Savelsbergh GJP, Mann DL. “What needs to be seen”: An exploration into the visual anticipation behaviour of different skill-level football referees while observing long passes on-field. Hum Mov Sci. 2022;85:102980. doi: 10.1016/j.humov.2022.102980 35908388

[15] Catteeuw P, Helsen W, Gilis B, Van Roie E, Wagemans J. Visual scan patterns and decision-making skills of expert assistant referees in offside situations. J Sport Exerc Psychol. 2009;31(6):786–97. doi: 10.1123/jsep.31.6.786 20384013

[16] Fasold F, Noël B, Wolf F, Hüttermann S. Coordinated gaze behaviour of handball referees: a practical exploration with focus on the methodical implementation. Mov Sport Sci/Sci Mot. 2018;(102):71–9. doi: 10.1051/sm/2018029

[17] Kittel A, Larkin P, Elsworthy N, Spittle M. Using 360° virtual reality as a decision-making assessment tool in sport. J Sci Med Sport. 2019;22(9):1049–53. doi: 10.1016/j.jsams.2019.03.012 30987883

[18] Catteeuw P, Gilis B, Jaspers A, Wagemans J, Helsen W. Training of perceptual-cognitive skills in offside decision making. J Sport Exerc Psychol. 2010;32(6):845–61. doi: 10.1123/jsep.32.6.845 21282841

[19] Świdwa J, Klatt S, Kantanista A. Gaze behavior and decision-making among handball referees: exploring gender and expertise differences. PeerJ. 2025;13:e19401. doi: 10.7717/peerj.19401 40386242 PMC12085115

[20] Spitz J, Wagemans J, Memmert D, Williams AM, Helsen WF. Video assistant referees (VAR): The impact of technology on decision making in association football referees. J Sports Sci. 2021;39(2):147–53. doi: 10.1080/02640414.2020.1809163 32794432

[21] Hancock DJ, Pizzera A. The psychology of sport officiating. Psychol Sport Exerc. 2025;80:102899. doi: 10.1016/j.psychsport.2025.102899 40447240

[22] Helsen W, Bultynck J-B. Physical and perceptual-cognitive demands of top-class refereeing in association football. J Sports Sci. 2004;22(2):179–89. doi: 10.1080/02640410310001641502 14998096

[23] Plessner H, Schweizer G, Brand R, O’Hare D. A multiple-cue learning approach as the basis for understanding and improving soccer referees’ decision making. Prog Brain Res. 2009;174:151–8. doi: 10.1016/S0079-6123(09)01313-2 19477337

[24] Nevill AM, Balmer NJ, Mark Williams A. The influence of crowd noise and experience upon refereeing decisions in football. Psychol of Sport Exerc. 2002;3(4):261–72. doi: 10.1016/s1469-0292(01)00033-4

[25] Dohmen TJ. The influence of social forces: evidence from the behavior of football referees. Econ Inq. 2008;46:411–24. doi: 10.1111/j.1465-7295.2007.00112.x

[26] Sors F, Grassi M, Agostini T, Murgia M. The sound of silence in association football: Home advantage and referee bias decrease in matches played without spectators. Eur J Sport Sci. 2021;21(12):1597–605. doi: 10.1080/17461391.2020.1845814 33131429

[27] Richlan F, Thürmer JL, Braid J, Kastner P, Leitner MC. Subjective experience, self-efficacy, and motivation of professional football referees during the COVID-19 pandemic. Humanit Soc Sci Commun. 2023;10(1):215. doi: 10.1057/s41599-023-01720-z 37192951 PMC10166025

[28] Anshel MH, Kang M, Jubenville C. Sources of acute sport stress scale for sports officials: Rasch calibration. Psychol Sport Exerc. 2013;14(3):362–70. doi: 10.1016/j.psychsport.2012.12.003

[29] Anshel MH, Weinberg RS. Sources of acute stress in American and Australian basketball referees. J Appl Sport Psychol. 1995;7:11–22. doi: 10.1080/10413209508406297

[30] Taylor AH, Daniel JV, Leith L, Burke RJ. Perceived stress, psychological burnout and paths to turnover intentions among sport officials. J Appl Sport Psychol. 1990;2(1):84–97. doi: 10.1080/10413209008406422

[31] Balch MJ, Scott D. Contrary to popular belief, refs are people too! Personality and perceptions of officials. J Sport Behav. 2007;30:3–20.

[32] Diotaiuti P, Falese L, Mancone S, Purromuto F. A structural Model of Self-efficacy in Handball Referees. Front Psychol. 2017;8:811. doi: 10.3389/fpsyg.2017.00811 28572783 PMC5435812

[33] Lirgg CD, Feltz DL, Merrie MD. Self-efficacy of sports officials: a critical review of the literature. J Sport Behav. 2016;39:39–50.

[34] Myers ND, Feltz DL, Guillén F, Dithurbide L. Development of, and initial validity evidence for, the referee self-efficacy scale: a multistudy report. J Sport Exerc Psychol. 2012;34(6):737–65. doi: 10.1123/jsep.34.6.737 23204357

[35] Karacam A, Adiguzel NS. Examining the relationship between referee performance and self-efficacy. Eur J Educ Res. 2019;8:377–82. doi: 10.12973/eu-jer.8.1.377

[36] Orhan BE, Karaçam A, Özdemi̇r AS, Çağlayan Tunç A. Examination of The Relationship Between Referee’s Emotional Regulation Status and Self-Efficiency. Mediterr J Sport Sci. 2022;5(Özel Sayı 1):19–27. doi: 10.38021/asbid.1199433

[37] Johansen BT, Haugen T. Coping with external stressors in handball and football elite refereeing: the relationship with referee efficacy. Scand J Sport Exerc Psychol. 2022;4:24–33. doi: 10.7146/sjsep.v4i1.129894

[38] Kiss B, Balogh L, Münnich Á, Csukonyi C. A sport-psychological diagnostic examination of young EHF handball referees with a focus on mental skills. J Phys Educ Sport. 2020;20:1984–95. doi: 10.7752/jpes.2020.04268

[39] Bloß N, Loffing F, Schorer J, Büsch D. Impact of psychological and physical load on the decision-making of top-class handball referees. Int J Perform Anal Sport. 2022;22(3):352–69. doi: 10.1080/24748668.2022.2061323

[40] Nurcahya Y, Rusdiana A, Hidayat Y, Sidik DZ, Tafakur M, Herdiansyah H. Effects of Mental Fatigue on Football Referee Decision Making. J Pendidik Jasmani Olahraga. 2025;10(1):186–92. doi: 10.17509/jpjo.v10i1.82038

[41] Kittel A, Larkin P, Elsworthy N, Spittle M. Video-based testing in sporting officials: A systematic review. Psychol Sport Exerc. 2019;43:261–70. doi: 10.1016/j.psychsport.2019.03.013

[42] Costa PT, McCrae RR. The five-factor model of personality and its relevance to personality disorders. J Pers Disord. 1992;6:343–59. doi: 10.1521/pedi.1992.6.4.343

[43] Zawadzki B, Strelau J, Szczepaniak P, Śliwińska M. Inwentarz osobowości NEO-FFI Costy i McCrae. Adaptacja polska. Podręcznik. Warszawa: Pracownia Testów Psychologicznych PTP; 1998.

[44] Dodt M, Fasold F, Memmert D. Personality profile of team handball referees at expert level. Ger J Exerc Sport Res. 2021;52(1):58–67. doi: 10.1007/s12662-021-00759-x PMC9281222 40479284

[45] Dodt M, Memmert D. The handball referee’s personality through the lens of players and coaches. Ger J Exerc Sport Res. 2023;54(3):462–75. doi: 10.1007/s12662-023-00926-2

[46] Shuai Y, Wang S, Liu X, Kueh YC, Kuan G. The influence of the five-factor model of personality on performance in competitive sports: a review. Front Psychol. 2023;14:1284378. doi: 10.3389/fpsyg.2023.1284378 38162969 PMC10756238

[47] Yang J-H, Yang HJ, Choi C, Bum C-H. Relationship between Athletes’ Big Five Model of Personality and Athletic Performance: Meta-Analysis. Behav Sci (Basel). 2024;14(1):71. doi: 10.3390/bs14010071 38275354 PMC10813302

[48] Allen MS, Greenlees I, Jones M. An investigation of the five-factor model of personality and coping behaviour in sport. J Sports Sci. 2011;29(8):841–50. doi: 10.1080/02640414.2011.565064 21500081

[49] Strelau J, Zawadzki B. The formal characteristics of behaviour—temperament inventory (FCB—TI): validity studies. Eur J Pers. 1995;9:207–29. doi: 10.1002/per.2410090304

[50] Strelau J. The regulative theory of temperament: current status. Pers Individ Dif. 1996;20:131–42. doi: 10.1016/0191-8869(95)00159-X

[51] Kovalchuk V, Mospan M. Psychological component of the basketball referee’s activity. J Phys Educ Sport. 2020;20(Suppl 1):522–8. doi: 10.7752/jpes.2020.s1078

[52] Voronova V, Romanyuk A, Shchepotina N, Kostiukevych V, Kovalchuk V, Kulchytska I, et al. Personal Factors of Referees in Football Influencing the Formation of Refereeing Styles. Phys Educ Theory Methodol. 2026;26(1):184–92. doi: 10.17309/tmfv.2026.1.18

[53] Leźnicka K, Starkowska A, Tomczak M, Cięszczyk P, Białecka M, Ligocka M, et al. Temperament as a modulating factor of pain sensitivity in combat sport athletes. Physiol Behav. 2017;180:131–6. doi: 10.1016/j.physbeh.2017.08.018 28844852

[54] Boldak A, Guszkowska M. Are Skydivers a Homogenous Group? Analysis of Features of Temperament, Sensation Seeking, and Risk Taking. Int J Aviat Psychol. 2013;23(3):197–212. doi: 10.1080/10508414.2013.799342

[55] Kaveshnikova EB, Alkharuf A, Pokrovskaya AV, Prikazchikova IV, Volpert BA. Features of the temperament of track and field athletics depending on their sports specialization. Curr Issues Sports Psychol Pedagogy. 2023;3(3):33–41. doi: 10.15826/spp.2023.3.77

[56] Bojkowski Ł, Tomczak M. Temperament structures and the effectiveness of individual play in football. Front Psychol. 2024;15:1376466. doi: 10.3389/fpsyg.2024.1376466 39176048 PMC11339529

[57] Chess S, Thomas A. Temperament and the concept of goodness of fit. In: Strelau J, Angleitner A, editors. Explorations in temperament: international perspectives on theory and measurement. New York: Plenum Press; 1991. p. 15–28.

[58] Berengüí R, Ruiz EJG de los F, Montero FJO, Marcos R de la V, Gullón JML. Optimism and Burnout in Competitive Sport. PSYCH. 2013;04(09):13–8. doi: 10.4236/psych.2013.49a2003

[59] Rainey DW. Stress, burnout, and intention to terminate among umpires. J Sport Behav. 1995;18:312–23.

[60] Nia ME, Besharat MA. Comparison of athletes’ personality characteristics in individual and team sports. Procedia Soc Behav Sci. 2010;5:808–12. doi: 10.1016/j.sbspro.2010.07.189

